# An alternative method for production of microalgal biodiesel using novel *Bacillus* lipase

**DOI:** 10.1007/s13205-014-0271-4

**Published:** 2015-01-07

**Authors:** Duraiarasan Surendhiran, Abdul Razack Sirajunnisa, Mani Vijay

**Affiliations:** Bioprocess Laboratory, Department of Chemical Engineering, Annamalai University, Annamalainagar, 608002 Tamilnadu India

**Keywords:** Biodiesel, *Nannochloropsis oculata*, Immobilized lipase, Encapsulation, Interesterification

## Abstract

In this study, enzymatic interesterification is carried out using encapsulated lipase as biocatalyst with methyl acetate as acyl acceptor in a solvent-free system. Lipase, isolated from a marine bacterial isolate, *Bacillus* sp.S23 (KF220659.1) was immobilized in sodium alginate beads. This investigation elaborated on the effects of various parameters, namely enzyme loading, temperature, water, molar ratio, reaction time and agitation for interesterification. The study resulted in the following optimal conditions: 1.5 g immobilized lipase, 1:12 molar ratio of oil to methyl acetate, 35 °C, 8 % water, 60 h reaction time, 250 rpm of agitation. With the standardized condition, the maximum conversion efficiency was 95.68 %. The immobilized beads, even after ten cycles of repeated usage showed high stability in the presence of methyl acetate and no loss of lipase activity. The microalgal biodiesel composition was analyzed using gas chromatography. The current study was efficient in using immobilized lipase for the interesterification process, since the method was cost-effective and eco-friendly, no solvent was involved and the enzyme was encapsulated in a natural polymer.

## Introduction

Alternative fuel has become a burgeoning global interest due to the deterioration and great consumption of fossil fuels leading to accumulation of greenhouse gases which paves the way for global warming (Su et al. 2007). Biodiesel (monoalkyl esters of long chain fatty acids) is a potential renewable biofuel and it is biodegradable and non-toxic, has no net carbon dioxide and is free from sulfur (Ali et al. 2011; Jeong and Park 2008; Kim et al. 2011; Li and Yan 2010). Generally, biodiesel is produced from food and oil crops using conventional methods (Tran et al. 2013); however, these sources cannot realistically satisfy the wide use of diesel fuel due to increasing population, which leads to serious land shortage and raises the issue of food security (Surendhiran and Vijay 2012). Microalgae have become a recent attraction because of their high oil content; they can be grown in wastewater, do not compete with food crops for arable land and water and give 20 times more biomass productivity rate than terrestrial crops (Ashokkumar and Rengasamy 2012; Chisti 2007; Lai et al. 2012; Mutanda et al. 2011; Pittman et al. 2011; Vandamme et al. 2010). Microalgae are photosynthetic microorganisms that utilize light, water and CO_2_ and accumulate intracellular lipids as storage materials (Xiao et al. 2010).

Currently, biodiesel is being produced by acid and alkali transesterification that results in conversion of triglycerides to fatty acid methyl esters in a shorter period (Jegannathan et al. 2010; Shao et al. 2008). Demerits of such methods include high energy input, elimination of salt, difficulty in recycling glycerol, soap formation and the need of wastewater treatment (Ban et al. 2002; Al-Zuhair et al. 2007; Bisen et al. 2010; Jeon and Yeom 2010; Kawakami et al. 2011; Ognjanovic et al. 2009; Rodriques and Zachia Ayub 2011; Yoshida et al. 2012). To overcome this problem, enzymatic production of biodiesel has become an alternative for biodiesel production, because the by-product glycerol can be easily recovered, salt and catalyst can be avoided, wastewater treatment is not required, it gives high production yield under milder conditions and is a eco-friendly process (Gharat and Rathod 2013; Gumbyte et al. 2011; Salum et al. 2010). One such enzyme used in biodiesel production is lipases. Lipases (triacylglycerol acylhydrolase, EC 3.1.1.3) are produced by microorganisms, plants and animals, out of which microorganisms are highly suitable for the large scale production (Antczak et al. 2009). Lipases are denoted as the most industrially important enzymes next to bacterial amylases. These are primarily used for catalyzing hydrolytic and ester-synthesizing reactions. It has been widely implemented in food and pharmaceutical sectors, and in various reactions such as acidolysis, aminolysis, alcoholysis, esterification and hydrolysis of triglycerides (Sivaramakrishnan and Muthukumar 2012).

However, the enzymatic production of biodiesel has not yet been commercialized due to the high cost of the enzyme. The problem can be overcome by immobilization of lipase by repeated use (Liu et al. 2012; Maceiras et al. 2009; Tamalampudi et al. 2008). In addition, transesterification is usually performed by methanol, but it deactivates the lipase enzyme resulting in poor yield of biodiesel. In this study, methyl acetate had been used as an acyl acceptor instead of methanol and the by-product was triacetin (triacetylglycerol) instead of glycerol. Triacetin (triacetylglycerol) is used mainly as gelatinizing agents in polymers and explosives and as additive agent in tobacco, pharmaceutical industries and cosmetics (Maddikeri et al. 2013). Presently, there has been a worldwide focus on the production of biodiesel in a solvent-free system using lipase. These systems are advantageous over solvent-aided transesterification, as separation processes, toxicity, flammability and high cost of organic solvents can be avoided.

## Materials and methods

### Culture conditions

*Nannochloropsis oculata* was obtained from CMFRI, Tuticorin, Tamil Nadu, India and cultivated in a 200 L photobioreactor (PBR) using sterile Walne’s medium. The filtered sterilized seawater was enriched with the required quantity of Walne’s medium containing: NaNO_3_, 100 g L^−1^; NaH_2_PO_4_·2H_2_O, 20.0 g L^−1^; Na_2_EDTA, 4.0 g L^−1^; H_3_BO_3_, 33.6 g L^−1^; MnCl_2_·4H_2_O g L^−1^, 0.36 g L^−1^; FeCl_3_·6H_2_O, 13.0 g L^−1^; vitamin B_12_, 0.001 g L^−1^, vitamin B_1_, 0.02 g L^−1^ and trace metal solution 1 ml. The trace metal solution contained: ZnSO_4_·7H_2_O, 21 g L^−1^; CoCl_2_ ·6H_2_O, 20 g L^−1^; (NH_4_)_6_Mo_7_O_24_·H_2_O, 9 g L^−1^; and CuSO_4_·5H_2_O, 20 g L^−1^. The medium was adjusted to pH 8 and autoclaved at 121 °C for 20 min. The filter-sterilized vitamins were added after cooling. Mixing was provided by sparging air from the bottom of the PBR and lighting was supplied by cool-white fluorescent light with an intensity of 5,000 lux under 12/12 light/dark cycle for 15 days. The medium was supplied with nitrogen for the first 4 days, after which the nutrients were added to PBR without nitrogen to create a nitrogen stress environment condition to produce more oil.

### Isolation and screening of lipase-producing bacteria from marine sediments

The lipase-producing bacteria were isolated from marine sediment at Parangipettai, a coastal area of Tamil Nadu, India. The samples were collected from sediment (5 cm depth) using a sterile container and immediately transferred to laboratory, serially diluted and spread on (medium composed of: peptone, 10 g L^−1^; NaCl, 5 g L^−1^; CaCl_2_·2H_2_O, 0.1 g L^−1^; agar–agar, 20 g L^−1^; Tween 20, 10 mL (v/v)) agar plates followed by incubation for 24 h at 37 °C. Lipase-producing bacteria produced a zone of clearance which was observed under UV transilluminator. Then the bacterial strain was isolated and subcultured using nutrient agar with 1 % olive oil and 3 % NaCl and subjected to studying morphological, cultural, spore production and biochemical characteristics.

### Gene sequence for molecular identification of the isolated strain

The molecular identification of the characterized culture was done by analyzing the genomic DNA. PCR analysis was performed with 16S rRNA primers: 27F (5′-AGA GTT TGA TCC TGG CTC AG-3′) and 1492R (5′- TAC GGT TAC CTT GTT ACG ACT T-3′). A volume of 25 µl reaction mixture for PCR was carried out using 10 ng of genomic DNA, 1X reaction buffer (10 mM Tris–HCl pH 8.8, 1.5 mM MgCl_2_, 50 mM KCl and 0.1 % Triton X-100), 0.4 mM dNTPs each, 0.5 U DNA polymerase and 1 mM reverse and forward primers each. The reaction was performed in 35 amplification cycles at 94 °C for 45 s, 55 °C for 60 s, 72 °C for 60 s and an extension step at 72 °C for 10 min. The sequencing of 16S amplicon was performed according to the manufacturer’s instructions of the Big Dye terminator cycle sequencing kit (Applied BioSystems, USA). Sequencing products were resolved on an Applied Biosystems model 3730XL automated DNA sequencing system (Applied BioSystems, USA). The 16S rRNA gene sequence obtained from the organism was compared with other *Bacillus* strains for pairwise identification using NCBI-BLAST (http://blast.ncbi.nlm.nih.gov/Blast.cgi) and multiple sequence alignments of the sequences were performed using Clustal Omega version of EBI (www.ebi.ac.uk/Tools/msa/clustalo). Phylogenetic tree was constructed by Clustal Omega of EBI (www.ebi.ac.uk/Tools/phylogeny/clustalw2_phylogeny) using neighbor joining method.

### Harvesting of cells and oil extraction

After the culture reaches the stationary phase at the 15th day (the culture was checked for growth every 24 h), the biomass was harvested using marine *B. subtilis* (MTCC 10619) to get thick microalgal paste as reported in our previous work (Surendhiran and Vijay 2014a, b). The dried biomass was subjected to oil extraction by Bligh and Dyer (1959) technique with slight modification. In brief, the biomass suspension was mixed with chloroform: methanol (1:2) ratio, vortexed for few minutes and incubated on ice for 10 min. Then, chloroform was added followed by addition of 1 M HCl and again vortexed for a few minutes. Finally, the whole suspension was centrifuged at a maximum speed of 12,000 rpm for 2 min. The bottom layer containing lipid was transferred into a fresh, previously weighed beaker. Chloroform was added to reextract the lipid from the aqueous sample. The solvent system was evaporated using a rotary evaporator at 30 °C. The final product, lipid, was collected in a screw cap vial and stored at room temperature.

### Fermentation of lipase production using isolated strain

Lipase production was carried out in a 250 ml Erlenmeyer flask using 100 ml basal medium containing 1 % olive oil, 0.2 % CaCl_2_·2H_2_O, 0.01 % MgSO_4_·7H_2_0, 0.04 % FeCl_3_·6H_2_O and 5 % NaCl, with 2 ml of starting inoculum. The contents were incubated for 48 h at 37 °C at 200 rpm and pH 7. After incubation, the culture was centrifuged at 10,000 rpm for 10 min at 4 °C. The supernatant of crude lipase was quantified using lipase assay and used for immobilization.

### Immobilization of crude lipase

Crude lipase (6 ml) was mixed with 14 ml of sodium alginate solution (2 %). The mixer was dripped into cold sterile 0.2 M CaCl_2_ using sterile syringe from a constant distance and was cured at 4 °C for 1 h. The beads were hardened by suspending it again in a fresh CaCl_2_ solution for 24 h at 4 °C with gentle agitation. After immobilization, the beads were separated by filtration and washed with 25 mM phosphate buffer (pH 6.0) to remove excess calcium chloride and enzyme. Then the beads were preserved at 18 °C using 0.9 % NaCl solution for future use (Kavardi et al. 2012; Vimalarasan et al. 2011).

### Lipase assay and protein determination

Lipase activity was determined for free and immobilized enzymes according to Burkert et al. (2004) and Padilha et al. (2012). The olive oil emulsion was prepared by mixing 25 ml of olive oil and 75 ml of 7 % Arabic gum solution in a homogenizer for 5 min at 500 rpm at room temperature. The reaction mixture containing 5 ml of emulsion, 2 ml of 10 mM phosphate buffer (pH 7.0) and 1 ml of the culture supernatant was incubated at 37 °C for 30 min in an orbital shaker. The reaction was stopped by addition of 15 ml of acetone–ethanol (1:1v/v), and the liberated fatty acids were titrated with 0.05 N NaOH. One unit of lipase activity was defined as the amount of enzyme that liberated 1 µmol of fatty acid per minute. The protein content in the crude enzyme was determined by Lowry et al.’s (1951) method with BSA as standard. The same procedure was done with 1 g of immobilized lipase to determine the percentage of immobilization according to Kavardi et al. (2012). The presence of protein in crude lipase was identified using sodium dodecyl sulfate–polyacrylamide gel electrophoresis (SDS-PAGE) with 12 % polyacrylamide gel.

### Determination of molecular weight of microalgal oil

According to Sathasivam and Manickam (1996), the saponification and acid value of microalgal oil were determined. The molecular weight of the oil was calculated as in Xu et al. (2006), the formula being:

$$M = \frac{168,300}{\text{ SV - AV}},$$

where *M* is the molecular weight of the oil, SV the saponification value and AV the acid value.

### Optimization of enzyme transesterification process by a solvent-free system

The enzymatic transesterification reaction was carried out in a 20 ml screw cap glass bottle. No solvent was added in this reaction. The reaction mixture consisted of 3 g of microalgal oil and 1 g of immobilized enzyme and methyl acetate. The oil to acyl acceptor (methyl acetate) was optimized ranging from 1:4, 1:6, 1:8, 1:10, 1:12 and 1:14. The effect of temperature was studied at various ranges of 25, 30, 35 and 40 °C. To investigate the effect of water, enzymatic transesterification was carried out by adding small amounts of water, at concentrations of 0, 2, 4, 6, 8 and 10 wt% of the total amount of the reaction mixture. The interesterification reaction was allowed for 48 h at a constant speed of 200 rpm. All the experiments were carried out in triplicate and the biodiesel yield was calculated according to Umdu et al. (2009).

### Gas chromatographic analysis of fatty acid methyl esters

Fatty acid methyl ester composition of biodiesel produced from *N. oculata* oil was analyzed by gas chromatography–mass spectrometry (GC–MS-QP 2010, Shimadzu) equipped with VF-5 MS capillary column (nonpolar, 30 mm length, 0.25 mm diameter and 0.25 µm film thickness). The column temperature of each run was started at 70 °C for 3 min, then raised to 300 °C and maintained at 300 °C for 9 min. GC conditions were: column oven temperature, 70 °C; injector temperature, 240 °C; injection mode, split; split ratio, 10; flow control mode, linear velocity; column flow, 1.51 ml/min; carrier gas, helium (99.9995 % purity); and injection volume, 1 µl. MS conditions were: ion source temperature, 200 °C; interface temperature, 240 °C; scan range, 40–1,000 m/z; solvent cut time, 5 min; MS start time, 5 min; end time, 35 min; ionization, EI (−70 eV); and scan speed, 2,000.

## Results and discussion

### Identification and characterization of lipase-producing marine bacterial isolate using 16S rRNA gene sequencing

Lipase-producing bacteria produced a zone of clearance around colonies with calcium precipitation due to hydrolysis of lauric, myristic, palmitic and stearic acids present in the medium containing Tween 20. The calcium precipitation was due to the formation of calcium salts and fatty acids released by the hydrolysis of lipase. The isolated marine sediment microorganism was analyzed by morphological and biochemical tests and found to be a Gram-positive rod, producing spores. The results obtained through various biochemical tests showed that the bacterium belonged to *Bacillus subtilis*. 16S rRNA gene sequencing was performed to identify the species and strain; culture confirmed that it was *Bacillus* sp.S23 (KF220659.1) through the phylogenetic tree.

### Quantification and characterization of microalgal oil

The oil content of *N. oculata* was calculated according to Suganya and Renganathan (2012) and the oil extraction yield was found to be 54.26 g (% w/w). The lipid concentration was defined as dry weight ratio of extracted lipids to biomass. The molecular weight of *N. oculata* oil was found to be 863.28, calculated from the acid value (0.58) and saponification value (195.53).

### Quantification of lipase assay and molecular weight determination of isolated lipase

**Fig. 1:**
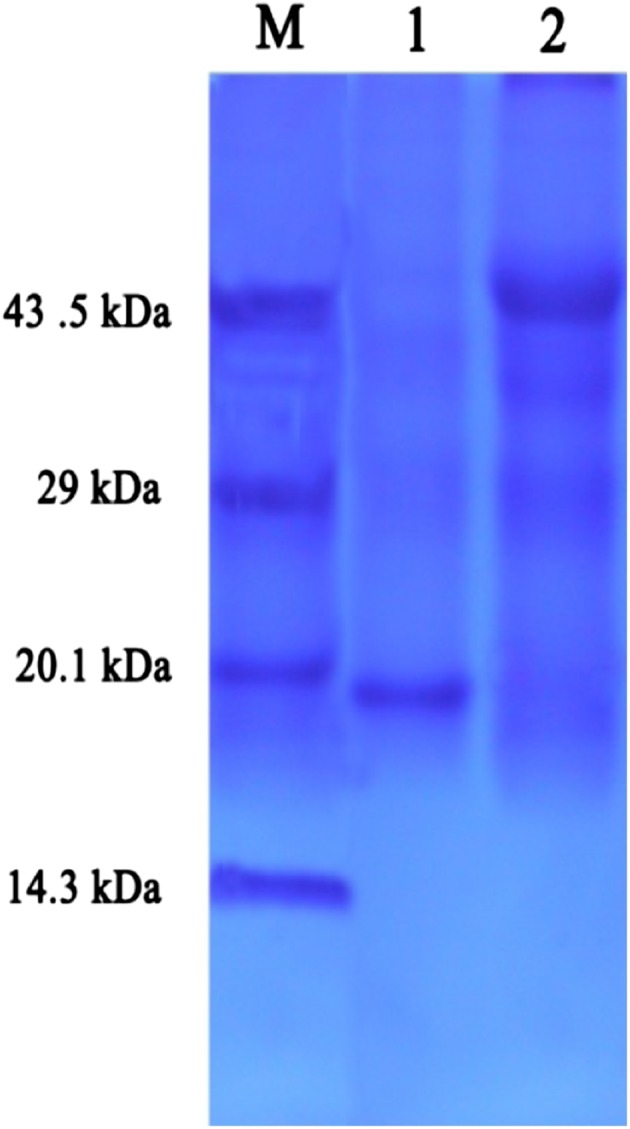
SDS-PAGE study of lipase isolated from marine bacteria *Bacillus* sp.S23 (KF220659.1). *Lane 1* protein marker, *lane 2* alum-purified lipase and *lane 3* crude lipase

The isolated marine *Bacillus* sp.S23 (KF220659.1) was used for lipase production at an optimum condition of 48 h at 40 °C at 200 rpm. 1 % olive oil was used for enhancing lipase production. The lipase activity from the culture supernatant was found to be 9.26 Uml^−1^. SDS-PAGE study revealed that the molecular weight of ammonium sulfate (40 % saturation)-purified extracellular lipase was nearly 20 kDa and crude lipase was around 45 kDa, which was confirmed with a standard marker (Fig. 1). Generally, genus *Bacillus* produces various types of lipases, based on molecular weight of protein ranging between 15 and 60 kDa. In the current study the lipase has low molecular weight of 20 kDa, which might be due to the conservative peptides such as Ala-His-Ser-Met-Gly in the protein (Sivaramakrishnan and Muthukumar 2012).

### Effect of enzyme loading

**Fig. 2:**
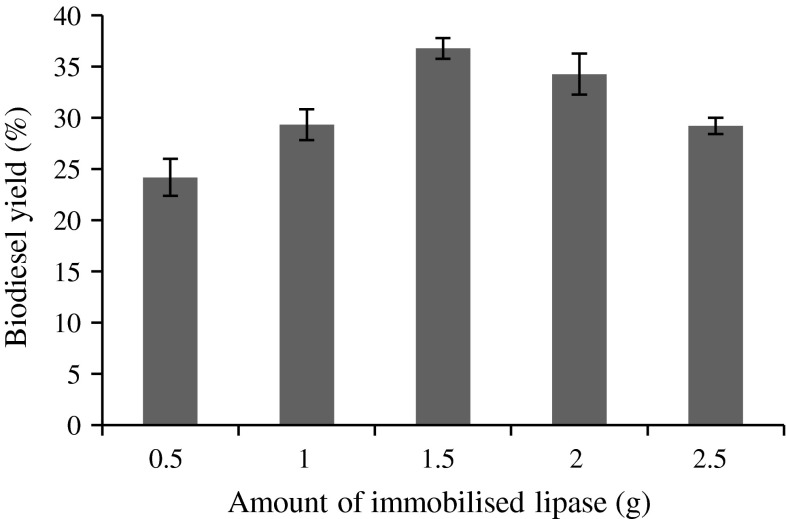
Effect of immobilized lipase loading on biodiesel yield (%). Reaction conditions: 1:4 oil/methyl acetate molar ratio, 30 °C, 200 rpm and 48 h

Effect of enzyme loading was studied to enhance transesterification in the range of 0.5–2.5 g. Figure 2 shows that the increasing enzyme loading resulted in increase in biodiesel yield when the load of immobilized beads was 1.5 g. The methyl ester yield was decreased at higher enzyme concentration. This is in agreement with Maceiras et al. (2009) and Jegannathan et al. (2010), who found that higher dosage of immobilized lipase results in lower yield of biodiesel. This is because the superfluous enzyme would unite and reduce the activity of lipase (Li and Yan 2010).

### Effect of oil and methyl acetate molar ratio

**Fig. 3:**
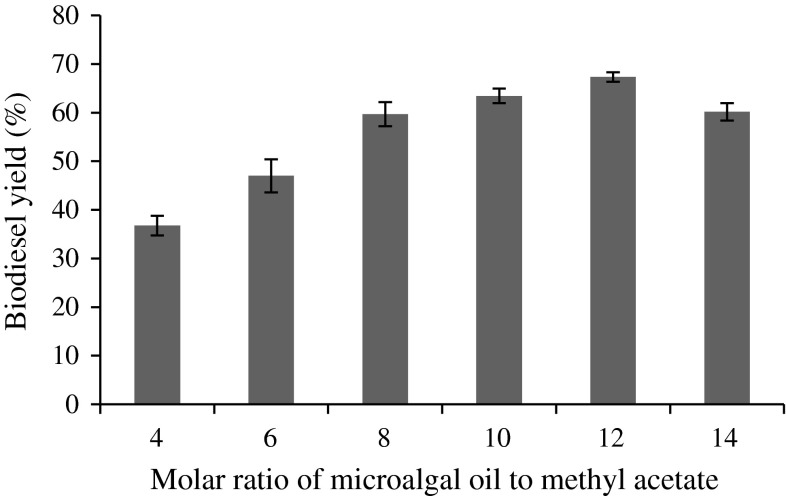
Effect of molar ratio of methyl acetate to microalgal oil on biodiesel yield (%). Reaction conditions: 1.5 g immobilized lipase, 30 °C, 200 rpm and 48 h

The effect of oil and methyl acetate ratio was investigated. It was found that 1:12 molar ratio of oil to methyl acetate gave maximum fatty acid methyl esters yield of 67.34 % at 48 h in the absence of any solvents, similar to previous study done by Ognjanovic et al. (2009). However, the biodiesel yield declined when the molar ratio was raised to 1:14 (Fig. 3), due to the excessive amount of methyl acetate that diluted the oil resulting in poor yield of fatty acid methyl esters. The conventional short chain alcohols such as ethanol and methanol inactivate the lipase enzyme when exceeding the 1:3 molar ratio. In support of this, Shimada et al. (1999) reported that inactivation of immobilized lipase Novozym 435 from *C. antarctica* occurred at a molar ratio of 1:5 of plant oil and methanol. In addition, during methanolic transesterification, the main by-product is glycerol, which is hydrophilic in nature and insoluble in oil, resulting in a decrease in the reactivity of immobilized lipase due to mass transfer resistance (Tran et al. 2013; Xu et al. 2003; Ruzich and Bassi 2011). Methyl acetate produces triacetylglycerol instead of glycerol, which does not inactivate lipase (Ruzich and Bassi 2011).

### Effect of temperature

**Fig. 4:**
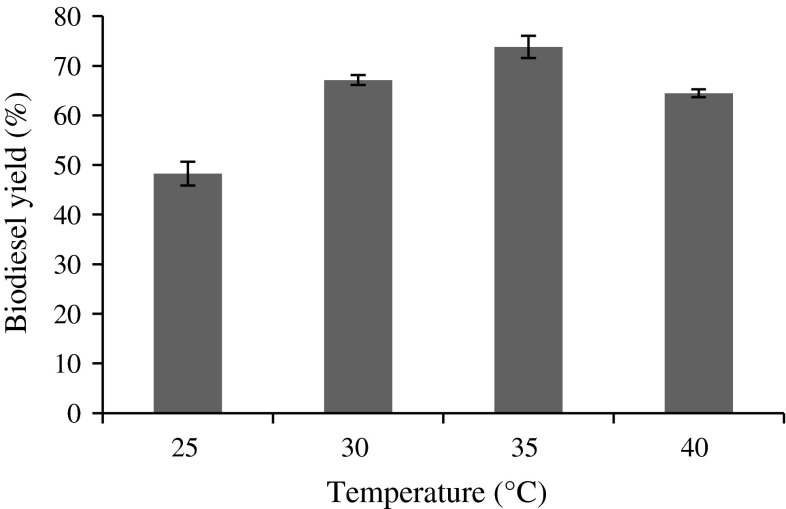
Effect of temperature on biodiesel yield (%). Reaction conditions: 1.5 g immobilized lipase, 1:12 oil/methyl acetate molar ratio, 200 rpm and 48 h

To study the effect of temperature on the enzymatic biodiesel process, the range studied was between 25 and 40 °C with an interval of 5 °C. The temperature was not allowed to exceed 40 °C, because sodium alginate dissolves at higher temperature. Tran et al. (2013) reported that FAME production decreased when the temperature increased to 50 °C for freshwater microalgae *C. vulgaris* ESP-31 by enzymatic transesterification. However, most of the enzymatic reaction does not require higher temperature (Jegannathan et al. 2010). In the current findings, 35 °C gave the highest yield of 73.79 % (Fig. 4), thereby reducing the energy consumption since higher temperature had not been used.

### Effect of water

**Fig. 5:**
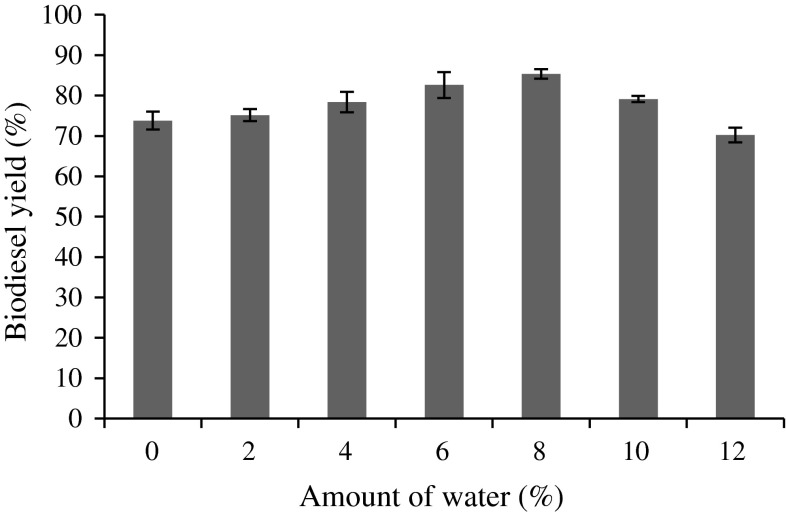
Effect of water on biodiesel yield (%). Reaction conditions: 1.5 g immobilized lipase, 1:12 oil/methyl acetate molar ratio, 35 °C, 200 rpm and 48 h

For biocatalyst-mediated transesterification, water acts as a key factor for enhancing the lipase activity by increasing interfacial area of oil–water droplets (Li and Yan 2010; Liu et al. 2012; Tran et al. 2012). Lipase activity generally depends on the availability of interfacial area (Dizge and Keskinler 2008). Li and Yan (2010) reported that exceeding the water content over 7 % of the total volume of the reaction mixture leads to decrease in the formation of FAME. However, in our study, there was no decrease of methyl esters until 8 % water content was achieved, which was due to the formation of triacylglycerol (triacetin) that did not disturb lipase activity. The highest yield of biodiesel was 85.36 % at 8 % water content. When the water content reached beyond 8 %, the yield was reduced (Fig. 5), due to the excess water content that reduced the transesterification reaction rate (Dizge and Keskinler 2008; Fukuda et al. 2006).

### Effect of reaction time on biodiesel yield

**Fig. 6:**
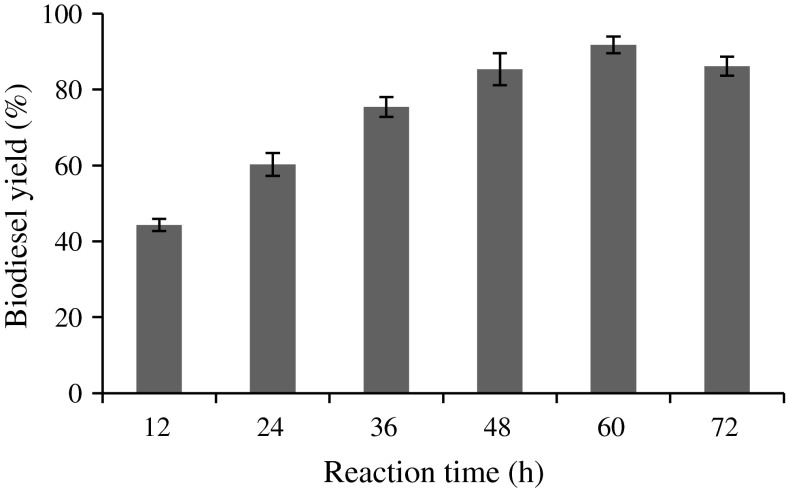
Effect of reaction time (%). Reaction conditions: 1.5 immobilized lipase, 1:12 oil/methyl acetate molar ratio, 4 % water (w/w) 35 °C and 200 rpm

Effect of reaction time was investigated in the range of 12–72 h. The optimized reaction time for conversion of microalgal oil to FAME by immobilized biocatalyst was found to be 60 h and the maximum yield was 89.48 % (Fig. 6). Beyond the maximal reaction at 60 h, a decrease in FAME was obtained. This is due to the increase in the water concentration during transesterification, which triggers the hydrolysis of the biodiesel (Jeong and Park 2008; Li and Yan 2010; Eevera et al. 2009; Leung et al. 2010).

### Effect of agitation speed

**Fig. 7:**
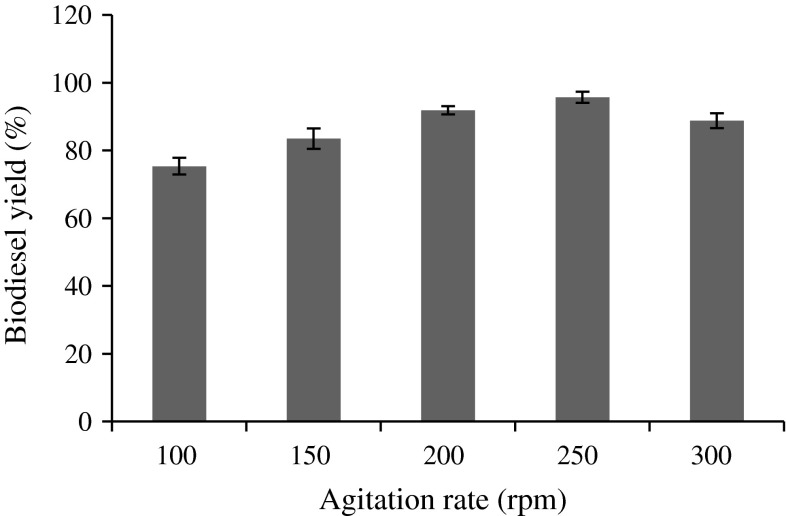
Effect of agitation on biodiesel yield (%). Reaction conditions: 1.5 g immobilized lipase, 1:12 oil/methyl acetate molar ratio, 4 % water (w/w) 35 °C and 60 h

Agitation is one of the important parameters in immobilized enzymatic transesterification. In the immobilization reaction system, the reactants need to diffuse from the bulk liquid to the external surface of the particle and then into the interior pores of the catalyst (Kumari et al. 2009). The effect of mixing on biodiesel production was conducted between 100 and 300 rpm with an interval of 100 rpm. Figure 7 shows the methyl ester production rate with the respective speed of agitation. The maximum yield of biodiesel was found to be 95.68 % at 250 rpm; thus, agitation enhances the rate of reaction. Agitation reduces the mass transfer resistance between oil and acyl acceptor and immobilizes lipase at the catalyzing interface, thus enhancing the reaction rate. On the other hand, when the speed reaches beyond 250 rpm, the biodiesel yield is decreased. This is due to the damage of the immobilized beads, leading to inactivation of lipase by mechanical agitation (Li and Yan 2010; Ognjanovic et al. 2009; Tran et al. 2012).

Xu et al. (2005) obtained a FAME yield of 67 % at 40 °C from refined soybean oil during interesterification reaction carried out at atmospheric pressure, with an oil to methyl acetate molar ratio of 1:12 and a reaction time of 36 h using Novozym (0.1 g of enzyme per 1 g of oil). Similarly, Usai et al. (2010) obtained 80 % of fatty acid methyl esters from olive oil with the reaction conditions of oil to methyl acetate molar ratio of 1:20 using immobilized lipase *Candida antarctica*. In our current study, the total biodiesel was 95.68 % at 1.5 g immobilized lipase, 1:12 molar ratio of oil to methyl acetate and at 35 °C.

### Reusability of immobilized enzyme

**Fig. 8:**
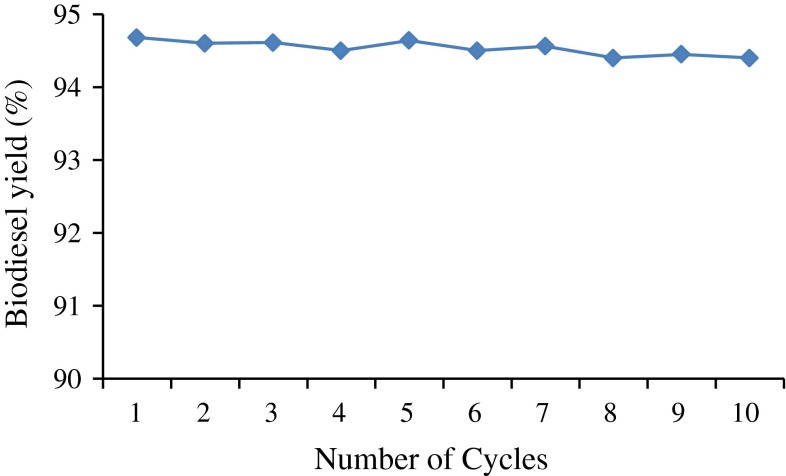
Reusability and stability of immobilized lipase on biodiesel yield (%). Reaction conditions: 1.5 g immobilized lipase, 1:12 oil/methyl acetate molar ratio, 4 % water (w/w), 35 °C, 250 rpm and 60 h

The main advantage of immobilized enzyme is its reusability. Reusability of enzyme is the important parameter to decide the possibilities of industrial-scale enzymatic biodiesel production (Gharat and Rathod 2013). Stability and reusability of immobilized lipase from marine *Bacillus* sp.S23 (KF220659.1) was investigated in this section. There was no significant loss of lipase activity even after immobilized enzyme beads were used for ten cycles (Fig. 8). As previously reported by Du et al. (2004), no enzyme loss was found even after 100 cycles of repeated usage in the presence of methyl acetate. When short chain alcohols (methanol and ethanol) are used as acyl acceptor, removal of glycerol from immobilized lipase must be carried out using large amounts of hydrophilic solvents, which is a cost-effective process and inhibits lipase activity. Thus, the current study indicates that immobilized lipase can be used for many repeated cycles in biodiesel production from microalgal oil with methyl acetate as acyl acceptor, which will minimize the cost factor in the overall process.

### Analysis of FAME

The fatty acid composition of biodiesel synthesized from *N. oculata* grown under nitrogen-depleted condition was analyzed and compared with FAME produced from nitrogen-repleted culture using GC (Table 1).

**Table 1:** Fatty acid composition of *N. oculata* FAME under nitrogen-repleted and nitrogen-depleted growth

Lipid number	Common Name	Systematic name	Molecular structure	Fatty acid (N^+^) %	Fatty acid (N^−^) %
C14:0	Myristic acid	Tetradecanoic acid	C_12_H_24_O_2_	9.86	8.94
C16:0	Palmitic acid	Hexadecanoic acid	C_16_H_32_O_2_	19.39	13.83
C18:0	Stearic acid	Octadecanoic acid	C_18_H_36_O_2_	10.76	9.79
C18:1	Oleic acid	9-Octadecenoic acid	C_18_H_34_O_2_	35.21	44.68
C18:2	Linoleic acid	9,12-Octadecadienoic acid	C_18_H_32_O_2_	8.15	6.92
C20:0	Arachidic acid	Eicosanoic acid	C_18_H_30_O_2_	16.62	15.84

*N* ^*+*^ presence of nitrogen, *N* ^*−*^ absence of nitrogen

**Fig. 9:**
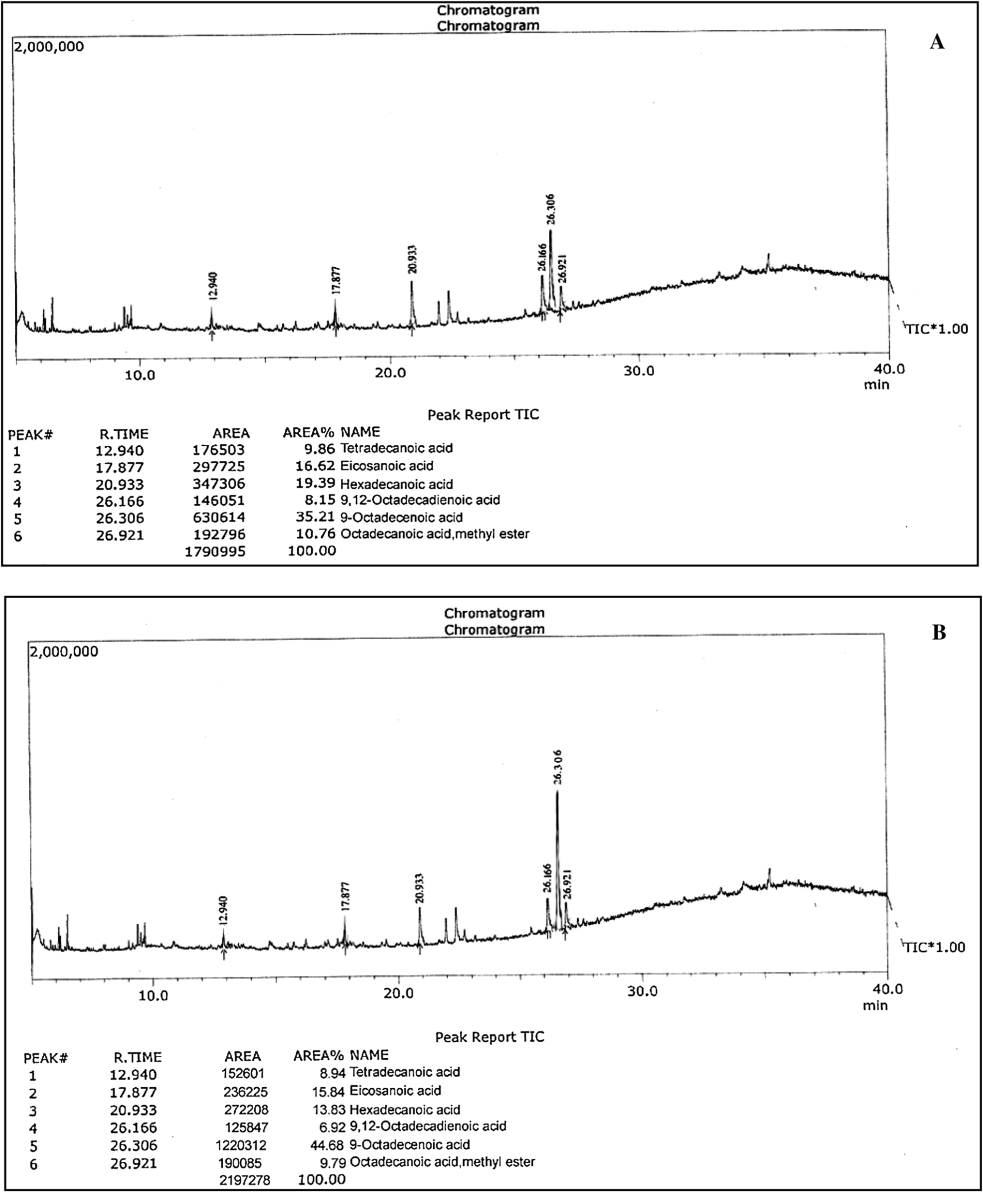
GC–MS chromatograms of *N.oculata* FAME under nitrogen-repleted (**a**) and nitrogen-depleted (**b**) growth

From the retention time obtained by GC, peak values were analyzed and observed as myristic acid (C14:0), palmitic acid (C16:0), stearic acid (C18:0), oleic acid (C18:1), linoleic acid (C18:2) and arachidic acid (C20:0), which were commonly found in biodiesel synthesized from *N. oculata* oil (Fig. 9). However, under nitrogen starvation condition, the lipid content not only doubled but also gradually changed the fatty acid composition of *N. oculata* oil (Surendhiran and Vijay 2014a, b; Huang et al. 2010; Widjaja et al. 2009). Moreover, in *N. oculata*, the oleic acid content increased from 35.21 to 44.68 % (Yoshida et al. 2012). This result was in better agreement with a previous study conducted by Zhila et al. (2005).

Unsaturated fatty acids have been reported to have a reasonable balance of fuel properties (Zheng et al. 2011). We have reported that the chain length of fatty acids in *N. oculata* was between C14 and C20 in our previously study (Surendhiran and Vijay 2014a, b). In a previous report, it was stated that fatty acids with maximum of C16 and C18 series were recognized as the most common components of biodiesel (Lin et al. 2012). Therefore, fatty acids from *N. oculata* were more applicable for producing a high quality of biofuel, since it contained a high content of C16 (palmitic acid) and C18 (oleic acid).

### Properties of biodiesel from *N. oculata*

The properties of *N. oculata* biodiesel synthesized through interesterification are listed in Table 2. The results were compared with that of diesel fuel and biodiesel from jatropha oil as stated by ASTM standard D6751. The final results revealed that no substantial variations were observed between the biodiesel properties of *N. oculata* and jatropha oil.

**Table 2:** Comparison of physio-chemical properties of biodiesel from *N. oculata* with petrodiesel and *Jatropha* biodiesel

Properties	Diesel fuel	Biodiesel from *Jatropha*	Biodiesel from *N. oculata*
Density (g/ml)	0.841	0.865	0.871
Kinematic viscosity (@ 40 °C)	1.9–4.5	5.2	5.71
Flash point (°C)	50–80	175	180
Fire point (°C)	78	136	153
Pour point (°C)	−6	−2	−4

## Conclusion

In this study, we have reported the conversion efficiency of marine microalga *N. oculata* oil to biodiesel using immobilized lipase in a solvent-free system with methyl acetate as the acyl acceptor. On studying the effects of different parameters influencing the process, an effective conversion rate of 95.68 % was observed. The optimized reaction conditions were 1.5 g immobilized lipase, 1:12 oil/methyl acetate molar ratio, 4 % water (w/w), 35 °C, 250 rpm and 60 h. The study revealed the potentiality of encapsulated lipase in transesterification due to its high stability and efficient activity after repeated usage. The present work is more advantageous than previous investigations, as it is a solvent-free system using only methyl acetate as the acyl acceptor, resulting in triacetin as the by-product that could be useful in various applications. The process also proved to be environmentally friendly and cost-effective due to the reusability of the immobilized beads.
